# Mild reproductive effects of the *Tityus bahiensis* scorpion venom in rats

**DOI:** 10.1186/1678-9199-20-4

**Published:** 2014-02-12

**Authors:** Ana Leticia C Dorce, Valquiria AC Dorce, Ana Leonor A Nencioni

**Affiliations:** 1 Laboratory of Pharmacology, Butantan Institute, Avenue Dr. Vital Brasil, 1500, São Paulo, SP CEP 05503-900, Brazil; 2 Post-Graduation Program of Disease Control, Coordination for the São Paulo State Public Health Secretary, São Paulo, SP, Brazil

**Keywords:** Scorpion venom, *Tityus bahiensis*, Pregnancy, Reproductive development, Embryofetotoxicity

## Abstract

**Background:**

Scorpion envenoming is a public health problem in Brazil, where *Tityus serrulatus* and *T. bahiensis* are considered the most dangerous scorpions. They are well adapted to urbanized environments, and there is an increasing probability of human exposure to these venoms, including during pregnancy. Not much is known about the effects of prenatal exposure to the venom, and no information is available to aid in the rational treatment of victims stung during pregnancy. Thus, this study aimed to investigate whether venom from the scorpion *T. bahiensis* administered once to pregnant female rats at a dose that causes a moderate envenomation may lead to deleterious effects on the reproductive performance of the dams and on the development of their offspring. This is the first work demonstrating that *T. bahiensis* venom, when administered experimentally to rats, alters maternal reproductive performance and the morphological development of fetuses. The venom was given to dams on the 5th (GD5) or on the 10th (GD10) gestational day. After laparotomy, on GD21, fetuses and placentas were counted, weighed and externally analyzed. The corpora lutea were counted. The sex and vitality of fetuses were evaluated, and each litter was then randomly divided for visceral or skeletal analyses. Data were analyzed by ANOVA followed by the Tukey-Kramer test and Fisher’s exact test. The significance level for all tests was set at p < 0.05.

**Results:**

GD5 group presented an increased number of pre-implantation losses. Weight gains in fetuses and placentas were observed in the GD5 and GD10 groups. Weights of the heart and lungs were elevated in GD5 and GD10 and liver weight in GD10.

**Conclusions:**

Moderate envenomation by *T. bahiensis* scorpion venom alters maternal reproductive performance and fetal development. However, these are preliminary results whose causes should be investigated more carefully in future studies.

## Background

Scorpions are dangerous animals distributed worldwide whose stings are considered a severe health problem. In Brazil, the dangerous scorpions belong to the genus *Tityus* (Arachnida class, Scorpiones order, Buthidae family) and comprise mainly the species *T. serrulatus*, *T. stigmurus* and *T. bahiensis*[1]. *T. serrulatus* (Lutz and Mello, 1922) is widespread in the southeastern region of Brazil and is not only responsible for most accidents but also the most severe cases mainly due to the high toxicity of its venom. The literature presents several subsequent studies concerning the venom and its toxins [1]. *T. bahiensis* can also be found in the same region and Lourenço *et al*. [2] showed that its venom presents some characteristics different from those of *T. serrulatus*, including convulsive properties. Such scorpion accidents happen frequently and with severity, especially among children and the elderly [3,4].

Human activity that leads to an increase in trash and waste attracts insects and spiders, the main prey of scorpions, while the possibility of occupancy of niches inside human dwellings causes an overlapping of the human and scorpion populations, thereby increasing the probability of incidents, where the possibility of envenomation of pregnant women is great [5].

Scorpion stings are characterized by intense local pain and, in some cases, by systemic effects. These include lung edema, partially due to severe cardiovascular alterations and to the release of catecholamines and kinins. Increasing pulmonary vascular permeability and complex respiratory arrhythmias such as tachypnea, hyperpnea, periodic respiration and respiratory paralysis, resulting from the stimulation of afferent visceral receptors or, alternatively, due to the action of the venom on the central nervous system, are also observed [2,6-11].

Scorpion venoms are composed of neurotoxins that affect sodium channels, or block potassium channels [12,13]. In both cases, the consequence is an increase in neurotransmitter release from neurons [14-16]. The scorpion sting produces tissue injury that can induce a systemic inflammatory response with the consequent release of cytokines [17]. It also stimulates the release of catecholamines, bradykinins and prostaglandins, which induce the release of IL-1 and IL-6 [18].

Although there have been several studies concerning the effects and the action mechanism of such venoms and their toxins, it is not well known whether they cause any harm to the offspring of mothers who have been stung during pregnancy, either accidentally or experimentally [19-24].

It is possible that the venom affects embryo development due to its effects on the release of chemical mediators such as neurotransmitters, growth factors and cytokine, all of which interfere in the course and regulation of pregnancy.

Studies have demonstrated that *T. serrulatus* scorpion venom evokes subtle alterations in reproductive parameters and fetal morphology [21,22] and that the venom of *T. bahiensis* elicits alterations in physical and reflexology development in the perinatal period and in the behavioral and neuronal development of offspring in adult life [23,24].

Since the respective venoms of *T. serrulatus* and *T. bahiensis* present some differences in the envenomation process, specific studies on *T. bahiensis* venom similar to those conducted on *T. serrulatus* venom are required to verify whether this venom causes some alterations in the reproductive performance and embryo development [21,22].

Thus, the present study was designed to investigate whether the venom of the scorpion *T. bahiensis* administered once to pregnant female rats at a dose that causes a moderate envenomation may lead to deleterious effects on the reproductive performance of dams and on the development of their offspring.

This is the first work that demonstrates impairment in the maternal reproductive development and in the morphology of fetuses after the injection of *T. bahiensis* scorpion venom during pregnancy.

## Methods

### Animals, mating and pregnancy diagnosis

Male (n = 15) and female (n = 30) Wistar rats (250–270 g) were housed under controlled conditions: 12-hour light/dark cycle (lights on at 7 a.m.) and air-conditioned at 22 ± 2°C, water and food were provided *ad libitum* throughout the experimental period.

After acclimatization for one week, two female rats were placed together with one male in the afternoon. On the morning of the following day, females showing evidence of mating (vaginal plug or vaginal smear with sperm cells) were randomly assigned to the study groups (n = 10). This day was recorded as gestation day 0 (GD0). During gestation, two dams were housed per plastic cage (40 × 50 × 20 cm). All the experimental procedures were conducted with prior permission of the institution’s Ethics Committee for Experiments on Animals (Protocol no. 513/08).

### Venom and solution

The venom, supplied by the Venom Commission of the Butantan Institute, had been obtained from the Arthropod Laboratory by electrical stimulation of the telson of mature *T. bahiensis* scorpions. It was lyophilized immediately after extraction and kept at –20ºC. Before use, it was dissolved in 1.46% (w/v) NaCl and maintained in ice. *T. bahiensis* venom is soluble in saline only at this concentration.

### Treatment

To mimic natural accidents, females received a single subcutaneous injection of 2.5 mg/kg of reconstituted *T. bahiensis* venom on the 5th (GD5) or 10th (GD10) gestational day. The control group received subcutaneous injection of 1.46% (w/v) NaCl on both days. The subcutaneous route was chosen since it may better simulate a naturally occurring scorpion sting. The venom dose used was chosen because, in previous studies performed in our laboratory, it caused a mild envenomation without rat death. The symptoms of this dose were compared in relation to local pain, respiratory perturbation and increased lacrimal and salivary secretions, which are mainly observed in moderate to severe scorpion poisoning.

### Reproductive parameters

The dams were weighed on GD0, GD5, GD10, GD16 and GD21. On GD21, females were deeply anesthetized with carbon dioxide (CO_2_) and submitted to laparotomy for sectioning of the ovaries and uterus. The gravid uterus was weighed. The uterine horns were cut, and the fetuses and their placenta were removed, weighed and examined for gross abnormalities. The vitality of fetuses was verified immediately after the withdrawal of the uterus (fetal movement after mechanical stimulation). The numbers of implantation sites, resorptions, and dead and live fetuses were recorded in both uterine horns. The ovaries were dissected and the number of corpora lutea was recorded. The pre-implantation losses (number of corpora lutea minus number of implantations/number of corpora lutea) and post-implantation losses (number of implantations minus number of live fetuses/number of implantations) were calculated.

After euthanasia, half of each litter was fixed in Bouin’s solution for subsequent macroscopic visceral examination by the Wilson serial sectioning method [25] in which the bodies are transversely sectioned with sharp blades into slices of 1 mm each, in order to analyze the defects and/or visceral anomalies of the following structures: palate, inner ear, spine, nasal cavity, nasal septum, retina, cornea, lens, hemisphere, ventricles (brain), salivary gland, thyroid, esophagus, trachea, thymus, heart, liver, kidney, bladder, ureter and gonads. The other half of the litter was eviscerated, fixed in 70% ethanol, cleared with potassium chloride and stained with alizarin red by the technique of Staples and Schenell [26].

Briefly, the fetuses were immersed in acetone and after 24 hours the organs were removed. The kidneys, liver, lungs and heart were weighed. The acetone was replaced with a solution of 0.8% KOH and alizarin. This solution was exchanged four times with a minimum interval of 24 hours between exchanges. Then, the solution was replaced with a bleaching solution (ethanol, glycerin, and benzyl alcohol PA). The extent of ossification was evaluated using the parameters proposed by Aliverti *et al*. [27]. Cranial bones, vertebrae, ribs, clavicles, sternum, metacarpus, metatarsus, and phalanges were analyzed as to the presence of anomalies. The pelvic girdle and the forelimbs and hindlimbs were examined with regard to the development of the long bones. The crown-rump lengths of fetuses were measured with a vernier caliper.

### Statistical analysis

Maternal reproductive parameters (number of implants, corpora lutea, resorptions, live fetuses, dead fetuses, pre-implantation loss and post-implantation loss) and the data on body or absolute and relative organ weight were analyzed by ANOVA followed by the Tukey-Kramer test. Skeletal and visceral anomalies and number of ossification centers were evaluated by the Fisher exact test. The significance level for all tests was set at p < 0.05.

## Results

### Maternal effects

A single dose of *T. bahiensis* venom (2.5 mg/kg) caused moderate systemic symptoms, namely increases in respiratory frequency and in salivary and nasal secretions, as well as local pain (increased sensitivity and vocalization to touch), and rigidity in hind limbs. These symptoms started approximately 5–10 minutes after the injection and persisted for 20–30 minutes. After this period, the rats returned to their normal state.

**Figure 1:**
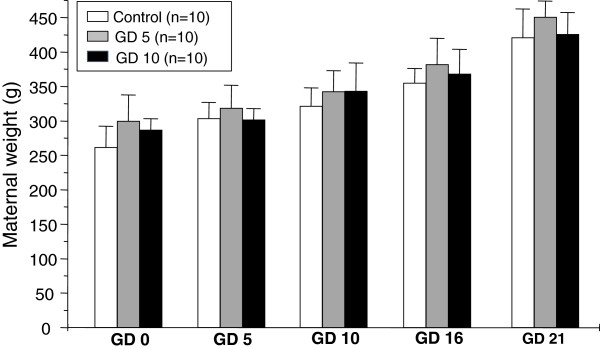
**Body weight gain (in grams) of dams treated with subcutaneous injection of 1.46% (w/v) NaCl (control group) or 2.5 mg/kg *Tityus bahiensis *scorpion venom on the 5th (GD5) and/or 10th (GD10) gestational day.** Values represent the mean ± SEM.

Treatment with venom on GD5 or GD10 did not affect maternal body weight gain during gestation (p > 0.05 – Figure 1). Neither maternal deaths nor other signs of toxicity, such as differences in food and water intake, were induced in the rats (Table 1). The general state and activity did not change in the treated groups, except in the period after the injection. The length of gestation was not altered in the experimental groups (births were not anticipated). The number of fetuses per litter and the sex ratios of the litters were not significantly different between the control and experimental groups (p > 0.05 – Table 2). Dead fetuses were not detected (Table 2).

**Table 1:** **Food and water intake of dams treated with *Tityus bahiensis *** **venom (2.5 mg/kg), on the 5th (GD5) or 10th (GD10) gestational day**

**Parameter**	**Pregnancy days**	**Control (n = 10)**	**GD 5 (n = 10)**	**GD 10 (n = 10)**	**p values**
Food intake (g/day)	1–7	19.4 ± 0.4	19.7 ± 0.7	20.0 ± 0.4	0.7326
	8–14	28.8 ± 0.5	28.6 ± 0.6	28.4 ± 0.5	0.8800
	15–21	30.0 ± 0.4	28.6 ± 0.6	28.5 ± 0.5	0.8050
Water ingestion (mL/day)	1–7	42.0 ± 1.7	47.5 ± 2.8	47.5 ± 2.6	0.1985
	8–14	57.0 ± 6.8	51.5 ± 3.2	51.0 ± 2.4	0.5949
	15–21	52.5 ± 3.7	52.5 ± 3.0	53.0 ± 3.7	0.8131

Data are expressed as the mean ± SEM. ANOVA followed by Tukey Kramer. n = number of dams.

**Table 2:** **Reproductive parameters of pregnant rats treated with *Tityus bahiensis *** **venom (2.5 mg/kg) on the 5th (GD5) or 10th (GD10) gestational day**

**Parameter**	**Control (n = 10)**	**GD5 (n = 9)**	**GD10 (n = 10)**	**p values**
Early resorptions/dam	0.36 ± 0.20	0.1 ± 0.10	0.2 ± 0.13	0.4133
Implantations/dam	14.2 ± 0.53	12.9 ± 0.69	13.0 ± 0.47	0.0547
Corpora lutea/dam	14.8 ± 0.44	14.7 ± 0.59	14.3 ± 0.42	0.7099
Live fetuses/dam	13.3 ± 0.59	12.9 ± 0.61	12.7 ± 0.61	0.7647
Dead fetuses/dam	0	0	0	–
Male fetuses/dam	6.7 ± 0.39	6.1 ± 0.75	6.5 ± 0.54	0.7766
Female fetuses/dam	6.5 ± 0.62	6.7 ± 0.62	6.4 ± 0.60	0.9544
Uterus weight (g)	99.5 ± 4.33	95.2 ± 6.62	94.8 ± 3.66	0.7662
Pre-implantation losses/dam	0.02 ± 0.01	0.13 ± 0.03*	0.09 ± 0.03	0.0321
Post-implantation losses/dam	0.06 ± 0.02	0.01 ± 0.01	0.03 ± 0.01	0.5353

Data are expressed as the mean ± SEM. *Significantly different from control; p < 0.05, ANOVA followed by Tukey Kramer. n = number of dams.

### Effects of *Tityus bahiensis* scorpion venom, administered on GD5 and GD10, on maternal reproductive development

**Figure 2:**
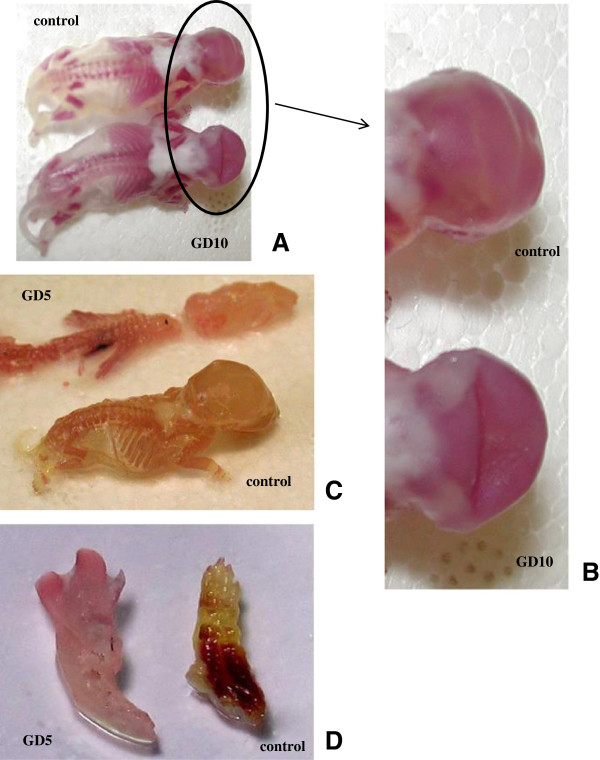
**Photograph of skeletal alterations. (A)** and **(B)** Deformity of the interparietal bone of the skull from GD10 group. **(C)** Longer fetuses from GD5 group (snout and head). **(D)** Detail of larger and malformed paw.

The GD5 group dams presented an increase in the number of pre-implantation losses (Table 2). The other reproductive parameters showed no significant alterations (Table 2). However, one dam that received the venom on GD5 showed three pre-implantation losses, three resorptions, and four dead fetuses. These fetuses were longer than the other fetuses from GD5; their snouts, paws and heads were larger than the ones of the other fetuses (Figure 2). However, this was the only litter presenting such a change, and it was not included in the statistical analysis. This type of anomaly was not verified in other control or experimental offspring. On GD10, no significant alteration was found (Table 2).

### Effects of *Tityus bahiensis* venom, administered on GD5 and GD10, on fetal morphology

The GD5 group presented significant weight increases in fetuses (Table 3) as well as placentas (Table 3), lungs (only absolute weight) and heart (Table 3). The liver and the kidneys did not show significant alterations (Table 3). There were no cases of anomalies or external malformations (Table 4). Macroscopic visceral analysis demonstrated that the organs had a normal appearance (Table 4). Skeletal analysis did not show any alterations in the fetal bone structure (Table 4) but the crown-rump distances were longer than in the control group (Table 3). The counts of offspring ossification centers did not differ significantly between the experimental groups and their controls.

**Table 3:** **Weights of fetuses, placentas and some internal organs of fetuses from dams treated with *Tityus bahiensis *** **venom (2.5 mg/kg) on the 5th (GD5) or 10th (GD10) gestational day**

**Weight**	**Control**	**n**	**GD5**	**n**	**GD10**	**n**	**p values**
Fetal weight (g)	5.10 ± 0.07	133	5.70 ± 0.04*	115	5.44 ± 0.04*	129	< 0.0001
Absolute placental weight (g)	0.49 ± 0.01	133	0.54 ± 0.01*	115	0.53 ± 0.01*	129	0.0010
Relative placental weight (%)	9.31 ± 0.29	133	9.96 ± 0.21	115	9.63 ± 0.14	129	0.6443
Absolute liver weight (g)	0.47 ± 0.01	66	0.49 ± 0.01	58	0.58 ± 0.01*	65	< 0.0001
Relative liver weight (%)	8.63 ± 2.06	66	8.78 ± 1.87	58	10.49 ± 1.34*	65	< 0.0001
Absolute lung weight (g)	0.14 ± 0.002	66	0.15 ± 0.003*	58	0.15 ± 0.002*	65	0.0107
Relative lung weight (%)	2.59 ± 0.05	66	2.76 ± 0.05*	58	2.79 ± 0.04*	65	0.0510
Absolute heart weight (g)	0.04 ± 0.001	66	0.06 ± 0.001*	58	0.05 ± 0.001*	65	< 0.0001
Relative heart weight (%)	0.83 ± 0.16	66	1.06 ± 0.16*	58	0.93 ± 0.14*	65	< 0.0001
Absolute kidney weight (g)^≠^	0.04 ± 0.001	66	0.04 ± 0.001	58	0.04 ± 0.001	65	0.5047
Relative kidney weight (%)	0.69 ± 0.02	66	0.67 ± 0.02	58	0.74 ± 0.02	65	0.0746
Crown-rump length (cm)	3.33 ± 0.02	66	3.47 ± 0.02*	46	3.35 ± 0.02	53	0.0002

Data are expressed as the mean ± SEM. *Significantly different from control; p < 0.05, ANOVA followed by Tukey Kramer. n = number of fetuses. ^≠^Kidneys were weighed in pairs.

**Table 4:** **Skeletal and visceral malformations or anomalies of fetuses from dams treated with *Tityus bahiensis *** **venom (2.5 mg/kg), on the 5th (GD5) or 10th (GD10) gestational day**

**Parameter**	**Control**	**GD5**	**p values**	**GD10**	**p values**
**External malformations**					
Number of affected fetuses	0/133	2/115	0.2140	0/129	–
Number of affected offspring	0/10	1/10	1.0000	0/10	–
**External anomalies**					
Number of affected fetuses	0/133	0/115	–	0/129	–
Number of affected offspring	0/10	0/10	–	0/10	–
***Skeletal malformations***					
Number of affected fetuses	0/66	0/46	–	0/53	–
Number of affected offspring	0/10	0/10	–	0/10	–
***Skeletal anomalies***					
Number of affected fetuses	10/66	11/46	0.3255	36/53*	< 0.0001
Number of affected offspring	4/10	5/10	1.0000	10/10*	0.0108
Deformity of interparietal bone	10/66	11/46	0.3255	36/53*	< 0.0001
Fused ribs	1/66	1/46	1.0000	2/53	0.5848
Shared vertebra	1/66	3/46	0.3036	0/53	1.0000
Rudimentary rib	0/66	0/46	–	1/53	0.4454
***Visceral malformations***					
Number of affected fetuses	0/67	2/57	0.2093	0/64	–
Number of affected offspring	0/10	1/10	1.000	0/10	–
***Visceral anomalies***					
Number of affected fetuses	1/67	2/57	0.5936	3/64	0.3579
Number of affected offspring	1/10	2/10	0.2727	3/10	0.3636
Cerebral hemorrhage	1/67	3/57	0.3328	3/64	0.3579

*Significantly different from control; p < 0.05, Fisher exact test.

In the GD10 group, significant increases were observed in the weights of fetuses as well as the placenta, liver, lungs and heart (Table 3). The kidney did not show significant alterations. There were no cases of anomalies or external malformations (Table 4). Macroscopic visceral analysis indicated that the organs had a normal appearance. In the skeletal analysis, no alterations were observed in the bone structure of the fetuses (Table 4). The number of ossification centers in sternum, metacarpals, metatarsals, and phalanges in the offspring of experimental and control groups did not differ significantly. No malformations of the pelvic girdle, forelimbs or hind-limbs were observed. Furthermore, no significant difference in the number of ossification centers was observed between groups. No wavy ribs, fused ribs, rudimentary rib or shared vertebrae were observed. The skull bones had a normal appearance, except for a deformity of the interparietal bone of the skull. All litters were affected (Table 4).

## Discussion

The fact that the scorpion species *T. serrulatus* and *T. bahiensis* are well adapted to urbanized environments augments the probability of exposure of humans and pets to these venoms, most perilously during pregnancy [21]. Not much is known about the effects of prenatal exposure to scorpion venom, and no information is available to aid in the rational treatment of victims stung during pregnancy [22]. Therefore, the present study aimed to assess the possible toxic effects of *T. bahiensis* venom on dams injected with this venom during pregnancy and on the prenatal development of their offspring.

A single non-lethal dose of the venom was employed to simulate a natural condition of envenomation. The intoxication of the dam on a more serious level was deliberately avoided so as not to compromise any positive result. The venom injection probably caused some pain to the animals, as evidenced by vocalization and attempts to run, which dissipated at 20–30 minutes after the application. This behavior was not observed in control dams.

The venom injection was carried out at two different stages of pregnancy, based on the rat development period, in accordance with Manson and Kang [28]. The 5th day of gestation corresponds to the end of the blastocyst implantation period in the uterus. Exposure to harmful substances in this period may lead to embryo lethality, with rare occurrence of teratogenesis. The 10th day of gestation coincides with the middle of the organogenesis period, which consists of a series of processes culminating in the formation of organs. Substances administered during this period may lead to teratogenesis, when the lesions caused by them enable the survival of the affected individual, or to embryo lethality, if the lesion is not compatible with embryo survival [29].

Studies in the literature reveal that some scorpion venoms are able to cause malformations and/or harmful effects on the offspring of experimental animals when the dams are inoculated during pregnancy. Ismail *et al.*[19] demonstrated that the venom of *Androctonus amoreuxi* caused a large number of fetal resorptions, underweight fetuses and skeleton defects when injected consecutively from the ninth to the twenty-first day of gestation in dams. He also found that the venom of *Buthus minax* caused skeleton malformation in goats and induced fetal resorption in pregnant women stung by this scorpion [19]. Scorpion venoms also have some effects on uterine contractility [30-32]. However, in an extensive review of the literature between the years of 1966 and 2002, Langley [33] found that no adverse consequences had been reported in either mother or fetuses in accidents occurring during pregnancy. Ben Nasr *et al.*[20] observed that two scorpion-envenomed pregnant patients developed intense pelvic pain and vaginal bleeding, but that neither maternal nor fetal death nor preterm fetal delivery was observed among twelve patients.

With regard to Brazilian scorpions, *T. serrulatus* venom has been demonstrated to cause some behavioral alteration in reproductive parameters and in fetal development when injected during pregnancy, whereas *T. bahiensis* venom causes some alterations in offspring development during the perinatal phase and in adult life [21-24].

Exposure to *T. bahiensis* venom did not affect the survival of pregnant females, the length of gestation, the number of fetuses per litter, or maternal body-weight gain. One of the obvious signs of maternal toxicity is a decrease in body-weight gain, which was not observed in our experiments [34].

Meanwhile, there was an increase in the number of pre-implantation losses at the end of the blastocyst implantation period (GD5 dams group), which can be explained by the fact that exposure to harmful substances in this period may be lethal [28]. Blastocyst implantation itself elicits an aseptic inflammatory-like reaction in the endometrium, in which cytokines such as IL-1, IL-11, TNF, LIF (leukemia inhibitory factor) and IGFBP (insulin-like growth factor protein) are considered to be significant modulators of the intrauterine immune phenomena [35,36]. Some works have demonstrated that scorpion venoms affect cytokines, an alteration that may be responsible for the losses observed [17,37-39].

An important finding was that a dam that had received the venom on GD5 presented three pre-implantation losses, three resorptions, and four dead fetuses. These fetuses were longer than the other fetuses from GD5; their snouts, paws and hearts were larger than the ones from the other fetuses. This might have resulted from an “all-or-none” effect, a phenomenon commonly occurring with scorpion venoms in which the effect may happen in a very strong fashion or not at all. Although this phenomenon has already been observed in several experiments in our laboratory and in clinical cases of envenoming, it lacks a clear explanation [22,40].

In relation to fetal development, weight alterations were observed in fetuses, placenta and some organs, in both gestational groups (GD5 and GD10).

Among the many metabolic processes performed by the mammalian placenta, several are considered essential for proper fetal development. For the fetus, the placenta is a multifunctional endocrine organ that is a combination of gastrointestinal tract, kidneys, lungs, liver, spleen and thymus. The placenta transports nutrients such as amino acids, vitamins, carbohydrates, lipids and minerals to the fetal circulation and is responsible for the metabolism of proteins, carbohydrates, lipids, prostaglandins, nucleic acids and steroid hormones [41,42]. An increase in placental weight may indicate an alteration in the metabolism of these substances, without, however, indicating whether such a change is benign or harmful [22]. Ben Nasr *et al*. [43] suggest that scorpion venom toxins may reach placental tissues and induce damage but there is no evidence that scorpion toxins bypass the placenta and reach the fetus [43].

The fetal weight increase observed in the present study may be a consequence of an increase in the weight of the organs or, alternatively, attributable to an augmentation of muscle or fat mass.

The role of factors regulating fetal growth, including growth hormone (GH), is important [44]. Studies across several species show that GH is an important determinant of litter size. It acts at all stages of litter-size development [44]. The interference of the venom with these factors may account for the increased weight of organs, but an accurate investigation is necessary to corroborate this finding.

During an envenomation, the lungs accumulate high levels of scorpion venom [45]. The respiratory system appears to be especially sensitive to the venom, since most fatal accidents are due to cardiovascular complications with consequent respiratory involvement [6,46]. Similarly, an increase in the pulmonary weight of fetuses was observed, confirming the sensitivity of the lungs to the venom. The mechanism by which the venom increases lung weight is not clear, but the development of the lung can be altered in many ways, including the release of glucocorticoids, which presumably occurs after contact with the venom [47]. Hmed *et al.*[48] demonstrated a similar increase after the injection of *Buthus occitanus tunetanus* scorpion venom, an effect related to defective placental function and to maternal hypertension and metabolic disorders.

A study in our laboratory demonstrated an increase in the weight of the lungs and placentas of fetuses whose mothers had received a dose of 1.0 mg/kg of *T. serrulatus* scorpion venom [22]. However, 3.0 mg/kg of the same venom had a similar effect on the liver, but the weights of the other organs were unaltered [49]. This leads us to hypothesize that a higher dose does not necessarily cause an increased effect. It may occur or not, independently of the dose administered. Although the increased weight of fetal organs is not traditionally related to fetal damage, the possibility that the observed result, an increase in liver, lung and heart weights, is a consequence of the venom cannot be ruled out. This effect could be better studied by histopathological examinations of the altered organs and by checking maternal metabolism in the same developmental period during which the present results were obtained.

In our study, there was no evidence of skeletal or visceral malformations in offspring of dams treated with *T. bahiensis* venom. On the other hand, an increase in skeletal size was observed in the GD5 group. Fetal development was not impaired; this was also demonstrated by counting the centers of ossifications, which disclosed no alterations. However, a deformity was observed in the interpariental bone in all the control and experimental fetuses but this deformity was augmented in the GD10 group. Anomalies in the skull bones were also described previously in the offspring of rats treated on GD10 with *Tityus serrulatus* scorpion venom [22,49]. Prenatal stress is responsible for alterations in skull ossification [50]. Actually, some alteration was verified in our experimental and control groups. Meanwhile, the GD10 group presented an alteration greater and significantly different from control group meaning that the venom may exert some effect but at this time, we do not have a sufficient experimental background to explain this result.

The observed visceral anomalies were very similar in amount and nature between the experimental groups and controls, which does not support the notion that the venom has a teratogenic effect. According to some authors, spontaneous anomalies and malformations eventually occur in laboratory rats [51,52]. Thus, it is unlikely that this occurrence had any link to prenatal exposure to the venom.

Based on the present results, we cannot determine whether the observed effects are due to the passage of some venom component to the offspring or whether it is just an indirect effect resulting from the response of the mother to the venom. However, Ismail *et al.*[19] reported that the teratogenic effect of the venom, observed after a prolonged exposure of pregnant rats to *A. amoreuxi* venom, appears to be the result of its metabolic effect and action on the body electrolytes of the dams, rather than a direct effect on the fetuses.

Despite our best efforts to avoid stress, either by means of the environmental conditions in which the animals were maintained and treated or by using a venom dose of apparently low maternal toxicity, stress was still a possible factor accounting for the results obtained. This fact is due to the inevitability of certain procedures such as the injection of the venom, which even at a mild dose always causes discomfort to the animals. Furthermore, we cannot rule out the possibility that although the venom had little effect on the dams, it had a more marked effect on the offspring, either by inducing stress or through a direct action.

Although it is not possible conclude that the effects observed are a direct consequence of the action of the venom, such effects indeed occurred after its administration. Additional studies are in progress to investigate the possible causes of the effects herein described.

## Conclusions

In conclusion, a moderate dose of *Tityus bahiensis* venom causes subtle changes in maternal reproductive development and in fetal development in rats. However, as some distinct alterations were observed, further studies are warranted which should include higher doses of the venom, other gestational days and the influence of cytokines on the development of the fetuses to elucidate the effects of the venom.

### Ethics committee approval

The present study was approved by the Ethics Committee for Experiments on Animals (protocol no. 513/08) of Butantan Institute.

## Competing interests

The authors declare that there are no competing interests.

## Authors’ contributions

ALCD and ALAN designed and developed the experiments, analyzed the results and prepared the manuscript for publication. VACD took part in planning experiments, analysis of results and preparation of manuscript for publication. All authors read and approved the final manuscript.

## References

[B1] Cologna CT Marcussi S Giglio JR Soares AM Arantes EC *Tityus serrulatus* scorpion venom and toxins: an overview Protein Pept Lett 2009 16 8 920 932 10.2174/092986609788923329 19689419

[B2] Lourenço GA Lebrun I Dorce VAC Neurotoxic effects of fractions isolated from *Tityus bahiensis* scorpion venom (Perty, 1834) Toxicon 2002 40 2 149 157 10.1016/S0041-0101(01)00202-1 11689236

[B3] Chippaux JP Goyffon M Epidemiology of scorpionism: a global appraisal Acta Trop 2008 107 2 71 79 10.1016/j.actatropica.2008.05.021 18579104

[B4] Chippaux JP Emerging options for the management of scorpion stings Drug Des Devel Ther 2012 6 165 173 22826633 10.2147/DDDT.S24754 PMC3401053

[B5] Lourenço WR Cloudsley-Thompson JL Cuellar O Von Eickstedt VRD Barraviera B Knox MB The evolution of scorpionism in Brazil in recent years J Venom Anim Toxins 1996 2 2 121 134

[B6] Freire-Maia L Peripheral effects of *Tityus serrulatus* scorpion venom J Toxicol Toxin Rev 1995 14 3 423 435 10.3109/15569549509019472

[B7] Sandoval MRL Dorce VAC Behavioral and electroencephalographic effects of *Tityus serrulatus* scorpion venom in rats Toxicon 1993 31 2 205 212 10.1016/0041-0101(93)90287-S 8456448

[B8] Dorce VAC Sandoval MRL Effects of *Tityus serrulatus* crude venom on the GABAergic and dopaminergic systems on the rat brain Toxicon 1994 32 12 1641 1647 10.1016/0041-0101(94)90322-0 7725331

[B9] Carvalho FF Nencioni ALA Lebrun I Sandoval MRL Dorce VAC Behavioral, electroencephalographic, and histopathologic effects of a neuropeptide isolated from *Tityus serrulatus* scorpion venom in rats Pharmacol Biochem Behav 1998 60 1 7 14 10.1016/S0091-3057(97)00407-3 9610917

[B10] Nencioni ALA Carvalho FF Lebrun I Dorce VAC Sandoval MRL Neurotoxic effects of three fractions isolated from *Tityus serrulatus* scorpion venom Pharmacol Toxicol 2000 86 4 149 155 10.1034/j.1600-0773.2000.d01-28.x 10815747

[B11] Nencioni ALA Lourenço GA Lebrun I Florio JC Dorce VAC Central effects of *Tityus serrulatus* and *Tityus bahiensis* scorpion venoms after intraperitoneal injection in rats Neurosci Lett 2009 463 3 234 238 10.1016/j.neulet.2009.08.006 19664683

[B12] de la Vega RC R Possani LD Overview of scorpion toxins specific for Na^+^ channels and related peptides: biodiversity, structure-function relationships and evolution Toxicon 2005 46 8 831 844 10.1016/j.toxicon.2005.09.006 16274721

[B13] de la Vega RC R Possani LD Current views on scorpion toxins specific for K^+^-channels Toxicon 2004 43 8 865 875 10.1016/j.toxicon.2004.03.022 15208019

[B14] Couraud F Jover E Tu AT Mechanism of action of scorpion toxins Handbook of Natural Toxins: Insect Poisons, Allergens and Other Invertebrate Venoms. Volume 2 1983 New York - Basel: Marcel Dekker 659 678

[B15] Possani LD Fletcher PL Fletcher MD Rode GS Mochca-Morales J Lucas S Coronas FV Alagon AC Martin BM Structural and functional characteristics of toxins purified from the venom of the Brazilian scorpion *Tityus serrulatus* Lutz and Mello Mem Inst Butantan 1992 54 2 35 52

[B16] Tan PT Ranganathan S Brusic V Deduction of functional peptide motifs in scorpion toxins J Pept Sci 2006 12 6 420 427 10.1002/psc.744 16432807

[B17] D’Suze G Moncada S Gonzalez C Sevcik C Aguilar V Alagon A Relationship between plasmatic levels of various cytokines, tumour necrosis factor, enzymes, glucose and venom concentration following *Tityus* scorpion sting Toxicon 2003 41 3 367 375 10.1016/S0041-0101(02)00331-8 12565760

[B18] Chaudry IH Stephan RN Harkema JM Dean RE Faist F, Ninneman J, Green D Immunological alterations following simple hemorrhage Immune Consequences of Trauma, Shock, and Sepsis 1989 Berlin: Springer 363 373

[B19] Ismail M Ellison AC Tilmisany AK Teratogenicity in the rat of the venom from the scorpion *Androctonus amoreuxi* (Aud. & Sav.) Toxicon 1983 21 2 177 189 10.1016/0041-0101(83)90002-8 6344336

[B20] Ben Nasr H Hammami TS Sahnoun Z Rebai T Bouaziz M Kassis M Zeghal KM Scorpion envenomation symptoms in pregnant women J Venom Anim Toxins Incl Trop Dis 2007 13 1 94 102 10.1590/S1678-91992007000100007

[B21] Barão AAS Bellot RG Dorce VAC Developmental effects of *Tityus serrulatus* scorpion venom on the rat offspring Brain Res Bull 2008 76 5 499 504 10.1016/j.brainresbull.2008.02.033 18534258

[B22] Cruttenden K Nencioni ALA Bernardi MM Dorce VAC Reproductive toxic effects of *Tityus serrulatus* scorpion venom in the rats Reprod Toxicol 2008 25 4 497 503 10.1016/j.reprotox.2008.04.011 18550329

[B23] Dorce ALC Bellot RG Dorce VAC Nencioni ALA Effects of prenatal exposure to *Tityus bahiensis* scorpion venom on rat offspring development Reprod Toxicol 2009 28 3 365 370 10.1016/j.reprotox.2009.04.008 19383539

[B24] Dorce ALC Dorce VAC Nencioni ALA Effects of *in utero* exposure to *Tityus bahiensis* scorpion venom in adult rats Neurotoxicol Teratol 2010 32 2 187 192 10.1016/j.ntt.2009.11.002 19945531

[B25] Wilson JG Wilson JC, Warkany J Methods for administering agents and detecting malformations in experimental animal Teratology: Principles and Techniques 1965 Chicago: University of Chicago Press

[B26] Staples RE Schenell VL Refinements in rapid clearing technic in the Koh-alizarin Red S method for fetal bone Stain Technol 1964 39 61 63 14106473

[B27] Aliverti V Bonanomi L Giavini E Leone VG Mariani L The extent of fetal ossification as an index of delayed development in teratogenic studies on the rat Teratololgy 1979 20 2 237 242 10.1002/tera.1420200208 524298

[B28] Manson J Kang YS Hayes AW Test methods for assessing female reproductive and developmental toxicology Principles and Methods of Toxicology 1989 2 New York: Raven 311 359

[B29] Rutledge JC Developmental toxicity induced during early stages of mammalian embryogenesis Mutat Res 1997 396 1–2 113 127 9434863 10.1016/s0027-5107(97)00178-4

[B30] Marei ZA Ibrahim SA Stimulation of rat uterus by venom from the scorpion *L. quinquestriatus* Toxicon 1979 17 3 251 258 10.1016/0041-0101(79)90215-0 473240

[B31] Meki AR Nassar AY Rochat H A bradykinin-potentiating peptide (peptide K12) isolated from the venom of Egyptian scorpion *Buthus occitanus* Peptides 1995 16 8 1359 1365 10.1016/0196-9781(95)02036-5 8745044

[B32] Mendonça M Luz MM Freire-Maia L Cunha-Melo JR Effect of scorpion toxin from *Tityus serrulatus* on the contraction of the isolated rat uterus Toxicon 1995 33 3 355 361 10.1016/0041-0101(94)00162-2 7638874

[B33] Langley RL A review of venomous animals and stings in pregnant patients Wilderness Environ Med 2004 15 3 207 215 10.1580/1080-6032(2004)15[207:AROVAB]2.0.CO;2 15473462

[B34] Chernoff N Rogers EH Gage MI Francis BM The relationship of maternal and fetal toxicity in developmental toxicology bioassays with notes on the biological significance of the “no observed adverse effect level” Reprod Toxicol 2008 25 2 192 202 10.1016/j.reprotox.2007.12.001 18242052

[B35] Fazleabas AT Kim JJ Strakova Z Implantation: embryonic signals and the modulation of the uterine environment - a review Placenta 2004 25 Suppl A S26 S31 15033303 10.1016/j.placenta.2004.01.014

[B36] Makrigiannakis A Minas V Kalantaridou SN Nikas G Chrousos GP Hormonal and cytokine regulation of early implantation Trends Endocrinol Metab 2006 17 5 178 185 10.1016/j.tem.2006.05.001 16698274

[B37] Magalhães MM Pereira MES Amaral CF Rezende NA Campolina D Bucaretchi F Gazzinelli RT Cunha-Melo JR Serum levels of cytokines in patients envenomed by *Tityus serrulatus* scorpion sting Toxicon 1999 37 8 1155 1164 10.1016/S0041-0101(98)00251-7 10400299

[B38] D’Suze G Salazar V Díaz P Sevcik C Azpurua H Bracho N Histopathological changes and inflammatory response induced by *Tityus discrepans* scorpion venom in rats Toxicon 2004 44 8 851 860 10.1016/j.toxicon.2004.08.021 15530967

[B39] Petricevich VL Scorpion venom and the inflammatory response Mediators Inflamm 2010 2010 903295 doi:10.1155/2010/903295 20300540 10.1155/2010/903295 PMC2838227

[B40] Ossanai LT Lourenço GA Nencioni ALA Lebrun I Yamanouye N Dorce VAC Effects of a toxin isolated from *Tityus bahiensis* scorpion venom on the hippocampus of rats Life Sci 2012 91 7–8 230 236 22771692 10.1016/j.lfs.2012.06.029

[B41] Gude NM Roberts CT Kalionis B King RG Growth and function of the normal human placenta Thromb Res 2004 114 5–6 397 407 15507270 10.1016/j.thromres.2004.06.038

[B42] Myllynen P Pasanen M Pelkonen O Human placenta: a human organ for developmental toxicology research and biomonitoring Placenta 2005 26 5 361 371 10.1016/j.placenta.2004.09.006 15850640

[B43] Ben Nasr H Serria H Chaker S Riadh B Zouheir S Kamel J Tarek R Khaled Z Some biological effects of scorpion envenomation in late pregnant rats Exp Toxicol Pathol 2009 61 6 573 580 10.1016/j.etp.2008.12.004 19185478

[B44] Waters MJ Kaye PL The role of growth hormone in fetal development Growth Horm IGF Res 2002 12 3 137 146 10.1016/S1096-6374(02)00018-7 12162995

[B45] Nunan EA Moraes MF Cardoso VN Moraes-Santos T Effect of age on body distribution of Tityustoxin from *Tityus serrulatus* scorpion venom in rats Life Sci 2003 73 3 319 325 10.1016/S0024-3205(03)00264-9 12757839

[B46] De Matos IM Talvani A Rocha OO Freire-Maia L Teixeira MM Evidence for a role of mast cells in the lung edema induced by *Tityus serrulatus* venom in rats Toxicon 2001 39 6 863 867 10.1016/S0041-0101(00)00225-7 11137547

[B47] Newman LM Johnson EM Johnson EM, Kochhar DM Abnormal lung function induced by prenatal insult Teratogenesis and Reproductive Toxicology. Volume 65 1983 New York: Springer 237 258

[B48] Hmed BN Riadh B Serria H Kamel J Khaled Z Embriotoxicity following repetitive maternal exposure to scorpion venom J Venom Anim Toxins incl Trop Dis 2012 18 3 317 324 10.1590/S1678-91992012000300009

[B49] Barão AAS Nencioni ALA Dorce VAC Embriotoxic effects of maternal exposure to *Tityus serrulatus* scorpion venom J Venom Anim Toxins incl Trop Dis 2008 14 2 322 337

[B50] Gonzalez PN Hallgrímsson B Oyhenart EE Developmental plasticity in covariance structure of the skull: effects of prenatal stress J Anat 2011 218 2 243 257 10.1111/j.1469-7580.2010.01326.x 21138433 PMC3042757

[B51] Kimmel CA Wilson JG Skeletal deviations in rats: malformations or variations? Teratology 1973 8 3 309 315 10.1002/tera.1420080311 4797551

[B52] Szabo KT Congenital Malformations in Laboratory and Farm Animals 1989 San Diego: Academic

